# Humeral shaft fractures: national trends in management

**DOI:** 10.1007/s10195-017-0459-6

**Published:** 2017-05-08

**Authors:** Bradley S. Schoch, Eric M. Padegimas, Mitchell Maltenfort, James Krieg, Surena Namdari

**Affiliations:** 1 Department of Orthopaedics and Rehabilitation, University of Florida, Gainesville, FL USA; 2 Department of Orthopedics, Thomas Jefferson University, Philadelphia, PA USA; 3 Department of Orthopaedic Surgery, Shoulder and Elbow Surgery, Rothman Institute, Thomas Jefferson University, 925 Chestnut St, 5th Floor, Philadelphia, PA 19107 USA

**Keywords:** Nationwide inpatient sample, NIS, Humerus fracture, Non-operative, Open reduction internal fixation

## Abstract

**Background:**

The incidence of humeral shaft fractures has been increasing over time. This represents a growing public health concern in a climate of cost containment. The purpose of this study is to analyze national trends in surgical management of humeral shaft fractures and determine factors predictive of surgical intervention.

**Materials and methods:**

Humeral shaft fractures were identified by the International Classification of Diseases, Ninth Revision, Clinical Modification codes 812.21 and 812.31 in the United States Nationwide Inpatient Sample from 2002 to 2011. Open reduction and internal fixation (ORIF) was identified by code 79.31 (ORIF, humerus). Other case codes analyzed were 79.01 (closed reduction without internal fixation), 79.11 (closed reduction with internal fixation), and 79.21 (open reduction without internal fixation). Multivariate regression analysis was utilized to determine predictive factors for utilization of ORIF.

**Results:**

27,908 humeral shaft fractures were identified. Utilization of ORIF increased from 47.2% of humeral shaft fractures in 2002 to 60.3% in 2011. Demographically, patients who underwent ORIF were younger (51.5 versus 59.7 years, *p* < 0.001; odds ratio 0.87 per decade of age). There were modest increases in ORIF usage with private insurance, open fracture, and hospital size, which persisted with multivariate regression analysis. Surprisingly, there was a tendency to shift from a slight increase in ORIF for males with the bivariate case to a slight preference for females in the multivariate case.

**Conclusion:**

Utilization of ORIF for humeral shaft fractures has been steadily increasing with time. Surgical intervention was more common with younger patients, female gender, private insurance, and larger hospital size. The increasing incidence of surgical management for humeral shaft fractures may represent a public health burden given the historical success of non-operative management.

**Level of evidence:**

IV.

## Introduction

Humeral shaft fractures represent 3% of all managed fractures and occur with an incidence of 13 per 100,000 per year [1, 2]. The incidence of these fractures has been increasing with the aging population [3]. These injuries occur in a bimodal age distribution affecting both young and old patients. Most patients are elderly (>65 years old), representing fragility-type fractures; however, these injuries also occur in younger patients (<30 years old) secondary to high-energy trauma [3]. Historically, non-operative management has been the preferred method for treating humeral shaft fractures, given the shoulder’s ability to compensate for angular and rotational malalignment [4, 5]. Sarmiento popularized non-operative management with a functional brace in 1977 after swelling had abated following 1–2 weeks in a coaptation splint [6, 7]. In contrast, both compression plating and intramedullary nailing were developed in an attempt to improve functional outcomes.

Recently, open reduction and internal fixation (ORIF) has become more prevalent, with analysis from Finland finding a two-fold increase in operative management between 1987 and 2009 [8]. However, in the United States, it is unclear how the incidence of ORIF has changed over time. Analysis of national trends may provide insight into changing surgeon and patient expectations. Additionally, understanding the national trends in management of humeral shaft fractures may clarify the public health burden that these injuries might represent. The purpose of this study is to analyze national trends in surgical management of humeral shaft fractures and determine any factors predictive of surgical intervention.

## Materials and methods

This study was conducted in the United States using the Nationwide Inpatient Sample (NIS). All humeral shaft fractures treated between 2002 and 2011 were identified using International Classification of Diseases, Ninth Revision, Clinical Modification (ICD-9-CM) codes. Patients were included if they received a diagnosis of 812.21 (closed fracture of humeral shaft) or 812.31 (open fracture of humeral shaft). The group was then subdivided into those treated non-operatively and those treated operatively. Fractures treated operatively were identified by ICD-9-CM code 79.01 (closed reduction without internal fixation), 79.11 (closed reduction with internal fixation), 79.21 (open reduction without internal fixation), or 79.31 (ORIF). Patients under the age of 18 were excluded, given the possible presence of open physes.

The NIS is currently the largest national payer database with the ability to follow inpatient hospitalizations. Information is captured from ~8 million patients and encompasses over 1000 hospitals, with results added on a yearly basis. Both federal and private hospitals are available for analysis, including smaller specialty hospitals. The annual survey has been estimated to represent 20% of all hospital discharges within the United States [9]. The Healthcare Cost and Utilization Project produces yearly statistical analyses to adjust for yearly variation in the NIS sampling [10, 11]. From this sample, demographic factors of age, gender, and ethnicity were recorded for each patient. Insurance variables and hospital size were also characterized.

Trends in utilization of ORIF over time were analyzed by Pearson’s correlation analysis. Multivariate analysis was utilized to determine predictive factors for utilization of ORIF. Microsoft Excel (2013; Redmond, WA, USA) and R [R Development Core Team (2008) R: a language and environment for statistical computing. R Foundation for Statistical Computing, Vienna, Austria] was used for all statistical calculations. Statistical significance was considered for a *p* value less than 0.05.

## Results

**Fig. 1:**
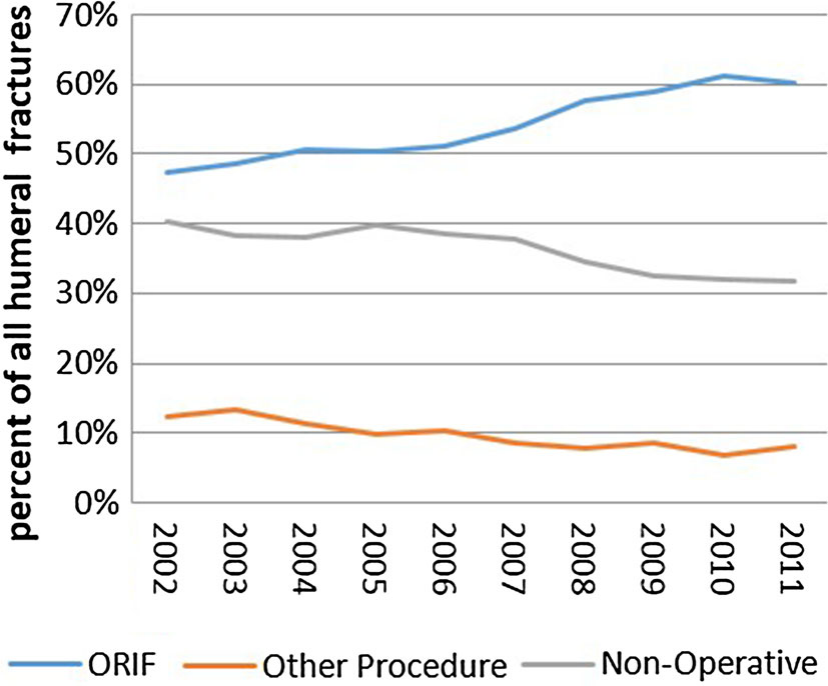
Annual trend of utilization of all treatment modalities for humeral shaft fractures

**Fig. 2:**
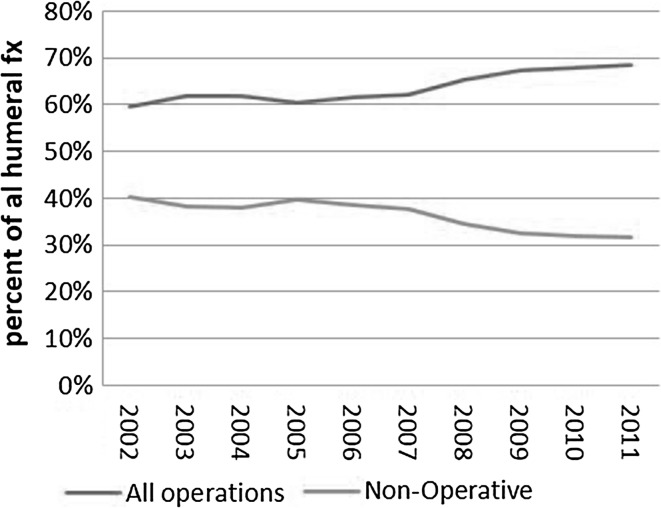
Annual trend of operative and non-operative management of humeral shaft fractures

Between 2002 and 2011, 27,908 humeral shaft fractures were identified through the NIS database. Using the assumption that the NIS database represents 20% of inpatient hospitalization discharges, Healthcare Cost and Utilization Project (HCUP) statistical corrections were used to estimate a national burden of 149,300 humeral shaft fractures over that time period [10, 11]. Of the 27,908 unique fractures identified in the sample, there were 15,142 (49.6%) who underwent ORIF, 4036 (14.5%) who underwent a surgical procedure other than ORIF (closed reduction without internal fixation, closed reduction with internal fixation, or open reduction without internal fixation), and 8730 (31.3%) who did not undergo surgical treatment. There were 2672 (9.6%) open fractures compared to 25,236 (90.4%) closed. In 2002, there were 2486 (8.9% of total) with 1174 (47.2%) treated by ORIF, 308 (12.4%) treated with a different surgical procedure, and 1004 (40.4%) treated non-operatively. By 2011, there were 3033 (10.9%) with 1828 (60.3%) treated by ORIF, 246 (8.1%) treated with a different surgical procedure, and 959 (31.6%) treated non-operatively. Logistic regression estimated that the probability for a humeral fracture patient receiving ORIF increased annually with an odds ratio (OR) of 1.07 per year [95% confidence interval (CI) 1.06–1.08], and that the odds for an operatively treated patient receiving ORIF increased with an OR of 1.10 per year (95% CI 1.08–1.11). The yearly trend for this is shown in Figs. 1 and 2.

**Fig. 3:**
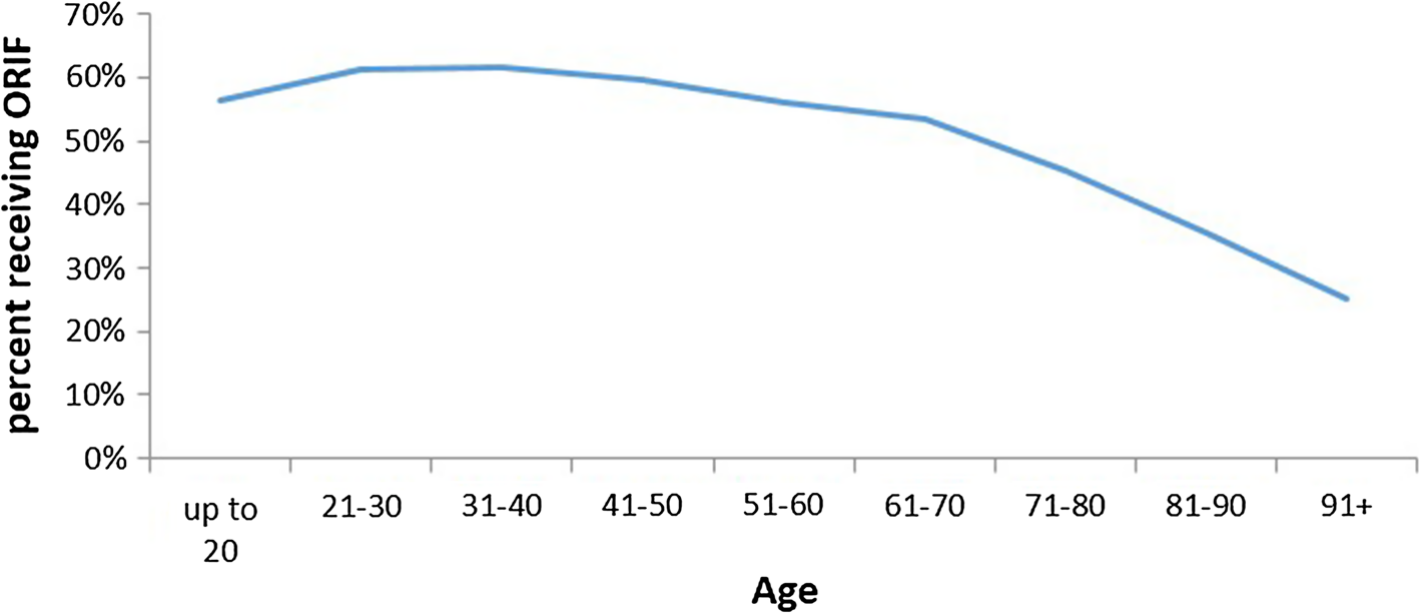
Utilization of operative intervention for humeral shaft fractures by age

Analyzing the entire study population of fracture, patients who underwent ORIF were younger (Fig. 3; 53.2 versus 61.2 years, *p* < 0.001), as seen in the trend in Fig. 3. Multivariate analysis adjusting for other factors showed odds of ORIF decreased with an OR of 0.87 (95% CI 0.85–0.88) per decade of age. When gender was analyzed in this multivariate regression analysis, males were slightly more likely to receive ORIF: 54.0% of males received ORIF versus 49.4% of females (*p* < 0.001). However, the multivariate analysis showed a slight preference for females getting ORIF (OR of 1.07, 95% CI 1.01–1.13, *p* = 0.026). With respect to insurance type, 42.2% of national government insurance for patients >65 years old (Medicare) and 55.0% of state government insurance for low-income patients (Medicaid) underwent ORIF compared to 59.4% of patients with private insurance. Comparing between insurance groups and correcting for multiple comparisons with the Holm–Bonferroni adjustment, Medicare had lower rates of ORIF than any other insurance group (*p* < 0.001), while private insurance had higher rates of ORIF than for self-pay (54.7%). Open fractures had higher rates of ORIF than closed fractures (65.1 vs 49.9%; adjusted OR 1.47, 95% CI 1.35–2.61, *p* < 0.0001). ORIF use also increased with the size of the hospital: 44.6% for small hospitals, 50.4% for medium, and 52.7% for large (*p* < 0.0001).

## Discussion

Union rates with non-operatively treated humeral shaft fracture have been reported between 67 and 98% [12–14]. Despite these rates, some patients are unable or unwilling to undergo non-operative management. Clinical union and removal of brace takes an average of 11.5 weeks with a range of 4–22 weeks with functional bracing compared with 6.3–9.8 weeks for intramedullary nailing and 8.9–10.4 weeks for compression plating [14–16]. Return to weight-bearing remains a function of bone quality and surgical fixation. Weight-bearing restrictions may be devastating to the elderly, who often require their arm to transfer or even weight-bear. In the younger patient, non-operative management may also delay their ability to return to work. In addition to functional limitations, functional bracing also carries a 1–9.5% risk of skin and soft tissue complications [17–19].

We demonstrate that surgical treatment of humeral shaft fractures in the United States has been increasing over time. The reason for this rise remains unclear, as numerous studies have reported satisfactory treatment with non-operative management. Complications following ORIF also occur at a similar rate to bracing, albeit with a different profile. Nerve palsy is the most common complication, reported in up to 7% of patients. Infection is also a common complication, affecting up to 3% of patients [20]. Possible reasons for increased ORIF utilization include a perceived quicker return to work, earlier initiation of shoulder and elbow rehabilitation, and avoidance of brace wear during the recovery period. While fixed-angle locked plating was introduced in 2005 and has been described for comminuted humeral shaft fractures and osteoporotic bone, the mainstay of treatment remains non-locked plating [21, 22]. Additionally, the increase in ORIF seen over time predated the introduction of locked plating, with no upward inflexion point seen over time. As a result, we do not believe that advancements in plate technology are responsible for the increased ORIF utilization described in this study. However, the development of intramedullary nailing for humeral shaft fractures does coincide with the timing of the increase in operative intervention [23, 24]. The increasing utilization of this technique may correlate with the observed trend.

Multiple predictive factors for operative intervention were identified. The first was younger age. We are unable to analyze other potential confounding factors which would make fixation more common in younger patients. However, older patients with osteoporotic bone and more medical comorbidities may be less willing to undertake or less likely to be recommended for surgery. Younger patients are also more likely to be involve in high energy poly-trauma [25], which is a relative indication for fixation of a humeral shaft fracture [26, 27]. Younger patients also have more demands to return to work and may not accept a longer duration of functional limitations. This result is in contrast with that of Matuszeqski et al. who showed that patients undergoing surgical fixation of humeral shaft fractures in the United States National Trauma Database were 3.5 years older than those treated non-operatively [28].

Open fractures were highly associated with fixation, which is a well-established indication for surgical intervention [23]. Patients with private insurance were also more likely to undergo operative intervention. This was an independent association on multivariate regression analysis and therefore not simply driven by Medicare patients being statistically older than privately insured patients. This finding is similar to a previous analysis that identified an association between surgery for upper extremity fracture and private insurance [29]. Additionally, previous analysis of supracondylar humerus fractures treated as outpatients in the pediatric population found that privately insured patients were nearly two and a half times as likely to return for surgical intervention than those with public or no insurance [30]. This independent association between lower surgical intervention rates and non-private insurance may be a barrier to care that pushes the treating surgeon to admit these patients rather than discharge for outpatient follow-up.

This study has multiple limitations. While the NIS database is a validated tool for studying population-based usage of medical resources, it remains limited by its capture of 20% of the population and relies on statistical modifiers to extrapolate to national projections. Additionally, as the NIS is an inpatient database, it is only able to capture those fractures that were admitted. Therefore, any patient treated in the emergency department with a coaptation splint, discharged for outpatient follow-up, and treated non-operatively would be missed. Similarly, any patient discharged for outpatient follow-up treated operatively, but in an outpatient surgical setting in which they were not admitted, would be missed. This study also does not explore the relationship between surgical fixation and discharge disposition, a relationship described by Matuszewski et al. [28]. Lastly, the NIS does not capture fracture classification or fixation type (plate size, locking/nonlocking), making analysis based on these variables impossible. Further outcomes-based studies are needed to identify the effect of increased operative fixation on patient outcomes in these groups.

In conclusion, humeral shaft fractures have continued to increase along with an aging population. Independent of this increased prevalence of humeral shaft fractures, the utilization of ORIF in the United States has also trended upwards in a similar manner. Predictive factors for operative ultization were identified as younger age, open fracture, and private insurance.
